# Cognitive Stimulation Therapy for older people with Dementia in Africa: A Scoping Review

**DOI:** 10.12688/openresafrica.14092.4

**Published:** 2026-01-16

**Authors:** Stephen Ojiambo Wandera, Edward Duncan, Monica Maria Diaz, David Otundo Ayuku

**Affiliations:** 1 Population Studies, School of Statistics and Planning, College of Business and Management Sciences, Makerere University, Kampala, Central Region, Uganda; 2 Behavioral Sciences and Mental Health, School of Medicine, Moi University, Eldoret, Uasin Gishu County, Kenya; 3 Nursing Midwifery and Allied Health Professions Research Unit, Pathfoot Building, University of Stirling, Stirling, Scotland, UK; 4 Department of Neurology, UNC School of Medicine, The University of North Carolina at Chapel Hill, Chapel Hill, North Carolina, USA

**Keywords:** Cognitive Stimulation Therapy, CST, sub-Saharan Africa, Dementia, Older Persons

## Abstract

**Background:**

Cognitive Stimulation Therapy (CST) is a non-pharmacological intervention developed for dementia that is useful in Africa but has not been studied widely. We reviewed the existing evidence regarding CST among older people living with dementia in Africa.

**Methods:**

A systematic literature search on CST among older people with dementia in Africa from 2003–2021 was done in MEDLINE (PubMed), CINAHL (EBSCOhost), and PsycINFO. A narrative approach was taken to chart, synthesize and interpret the data using Microsoft Excel.

**Results:**

After removing duplicates using Endnote, a total of 122 studies were retained and screened first by title, then abstract, and finally by full text. Six articles matched the inclusion/exclusion criteria. CST has been adapted and piloted in two African countries (Nigeria and Tanzania). CST studies in Africa indicate improvements in clinical outcomes including cognition and quality of life. Although there are some barriers to overcome, CST may be applicable to an African context.

**Conclusions:**

CST is feasible, adaptable, and acceptable in the African countries it has been implemented in. Some cultural barriers, such as religious affiliation and respect for older people, should be overcome. Further research is needed to further evaluate the efficacy of CST in various African contexts.

## Introduction

Globally, about 9% of the population is aged 65 years and older, projected to increase to 17% by 2050 ^1, 2^. In sub-Saharan Africa, adults aged 60 years and older are expected to reach 670 million by 2030, with 10% of the population aged 60 years or older by 2050 ^3– 5^. Older persons often experience multimorbidity from non-communicable diseases, including dementia, which causes a progressive decline in cognition and behavior ^5– 7^. Alzheimer's disease, the most common type of dementia, accounts for 60–70% of new cases ^7– 9^. The global proportion of people living with dementia (PWD) ranges between 2% and 9%, with 27 million (60%) in low- and middle-income countries (LMICs), projected to increase to 81 million by 2040 and 150 million by 2050 ^10– 12^. Risk factors for dementia include age, sex, low education, hypertension, stroke, poor socioeconomic status, vascular disease, diabetes, tobacco use, obesity, harmful alcohol use, physical inactivity, depression, social isolation, and HIV ^13– 15^. Dementia impacts older adults physically, psychologically, socially, and economically ^16, 17^, leading to declines in cognition, quality of life, and independence, and also affects caregivers through caregiver burden ^8, 12, 16– 19^.

Currently, there are significant resource limitations to diagnose and treat dementia in sub-Saharan Africa. An ethnographic study in a tertiary care hospital in Nairobi revealed that while their dementia diagnostic pathway aligns with best practices, most public hospitals in Kenya lack the necessary diagnostic equipment and trained staff, limiting access to timely care ^20^. Barriers to effective diagnosis and care include the absence of dementia policies, limited healthcare provider expertise, high costs, and sociocultural factors like stigma, emphasizing the need for government action to improve awareness, training, and resources. Another exploratory qualitative study involving 25 imaging personnel and physicians in Uganda revealed that while brain MRI is recognized as crucial for diagnosing dementia, its use is limited by high costs, restricted access, mechanical failures, patient-related issues, and interpretation difficulties ^21^. A recent review article highlighted Alzheimer's disease in Africa, focusing on genetic and modifiable risks, diagnostic and treatment challenges, and the need for policy responses. Key barriers to dementia diagnosis included diagnostic infrastructure shortcomings, resource limitations, and knowledge gaps, necessitating a multipronged strategy to improve diagnostic capacities, treatment availability, specialist training, public awareness, and coordinated policymaking ^22^.

Management of dementia is twofold: pharmacological treatments and non-pharmacological or psychosocial interventions ^23^. Pharmacological treatments delay the progression of the disease. Conversely, psychosocial interventions improve cognition and quality of life and reduce caregivers’ burden ^19, 24^. Cognitive Stimulation Therapy (CST) is an evidence-based non-pharmacological intervention developed for dementia ^10^. Cognitive Stimulation Therapy (CST) is the best non-pharmacological intervention developed for people with dementia ^10^.

The CST is recognized as a cost-effective, evidence-based, and gold-standard treatment for dementia among older people ^18, 25^. It is a group-based psychosocial intervention for people with mild to moderate dementia ^26– 31^. Recent studies demonstrated that CST is as effective than currently available dementia medications ^32^. Since CST has demonstrated efficacy in delaying the cognitive decline expected in mild-to-moderate dementia, the CST is positioned to become a global standard of care for dementia in light of limited access to medications in sub-Saharan Africa.

### Rationale and study objectives

The rationale of this scoping review was that despite the CST’s efficacy, there is limited evidence of synthesized data on its applicability to older persons with dementia in Africa. Therefore, we aimed to synthesize the available evidence of studies conducted on CST within sub-Saharan Africa. Specifically, we aimed to describe the 1) characteristics of CST studies; 2) primary outcomes of CST; 3) Adaptations/modifications of CST delivery in Africa; and 4) barriers and facilitators of CST within African contexts ^33, 34^. 

## Methods

### Protocol registration

The protocol for this scoping review was not registered. Because Prospero does not register scoping reviews. Also, the JBI does not make it mandatory to register protocols for scoping reviews ^35^.

### Study design

We conducted a scoping review of the original version of CST among older persons in Africa ^26, 28, 30^. We focused on studies that administered the original version of the CST ^26, 28, 30^. CST consists of 14 sessions of 45–60 minutes duration each, occurring twice a week for seven weeks ^26, 36^. The 14 sessions of CST include topics that cover physical games, sound (e.g. known folk songs), childhood memories (e.g. childhood home, traditional foods), food (e.g. local dishes), current affairs, faces/scenes, word association, being creative, categorizing objects, orientation, using money, number and word games, and team quizzes ^26, 30, 37^. CST is beneficial in the African context because it helps caregivers and is an alternative to dementia medications that are not accessible in rural Africa. The CST intervention was developed for international use ^29, 38, 39^. It was adapted for Africa in 2016 ^37^ and has been piloted in Tanzania, and Nigeria ^29, 40– 42^.

### Eligibility criteria

We used the **Population, Concept, and Context (PCC) framework** to guide the reporting of the scoping review ^43^. The study **population** was older people (age 50 years and older) living with dementia. The WHO recommends that for African countries, we define older persons as those 50 years and older ^44^. Several studies from the IN-DEPTH network have used this definition in their studies ^45– 48^. The **concept** or the intervention was CST. The outcomes for the scoping review included: improvement in cognition, quality of life, and feasibility, adaptability, implementation, and barriers and facilitators to CST. The **context** was sub-Saharan Africa.

The **inclusion** criteria were papers that reported studies of CST; participants were older people with dementia in Africa; papers were peer-reviewed and were published in English and were in the timeline between 2003 and 2021. The year 2003 is when the first article on CST was published ^26^. There was an earlier (1994) publication on cognitive stimulation for dementia patients, but it was not the CST ^49^.

Papers were **excluded** if they were studies solely conducted outside of Africa; were not published in peer-reviewed journals; we excluded approaches or procedures unrelated to CST. In addition, we excluded systematic reviews ^10^.

### Information sources

A systematic literature search on CST among older people with dementia in Africa from 2003 to 2021 was conducted for MEDLINE (via PubMed), CINAHL (via EBSCOhost), and PsycINFO (via EBSCOhost) ^50^. The databases searched were based on two previous studies ^7, 50^. Internationally recognized principles for searching, screening, and appraising results and for conducting a scoping review were followed ^51^.

### Search strategy

Table 1 presents the databases searched in February and March 2021. Search terms were adapted and refined from previous reviews ^6, 7, 10, 52, 53^ and by consensus between the authors (SOW and ED). Search terms included cognitive stimulation therapy (CST), dementia, and Africa. The search terms were combined using Boolean Operators (OR, AND).

**Table 1.** Databases Searched.

Database type	Database	Period covered
Nursing and Allied Health Services	Cumulative Index of Nursing and Allied Health Literature (CINAHL)	1937 to present
Life Sciences and Biomedical Literature	MEDLINE	1870 to present
Life Sciences and Biomedical Literature	PubMed	1996 to present
Abstracts in the Field of Psychology	PsycInfo	1887 to present

Supplementary Table 1 ^54^ presents the full search strategy for the scoping review. The search terms used to identify African nations were adapted from another study ^55^. The adaptation involved removing the “tw” (“(text word” search searching in the title and abstract fields) from the search terms, as this field search restricted results ^56^.

### Selection of sources of evidence

Relevant studies were identified by searching electronic databases, reference lists, and key journals, and consulting Prof. Spector, lead developer of CST ^57^. The databases searched were based on two previous studies ^7, 50^: MEDLINE (via PubMed), CINAHL (via EBSCOhost), and PsycINFO (via EBSCOhost) ^50^. Reference lists of identified studies were searched to identify additional studies. Searches were limited to English between 2003–2021. Articles were selected using the set inclusion and exclusion criteria ^57^.

**Figure 1.**
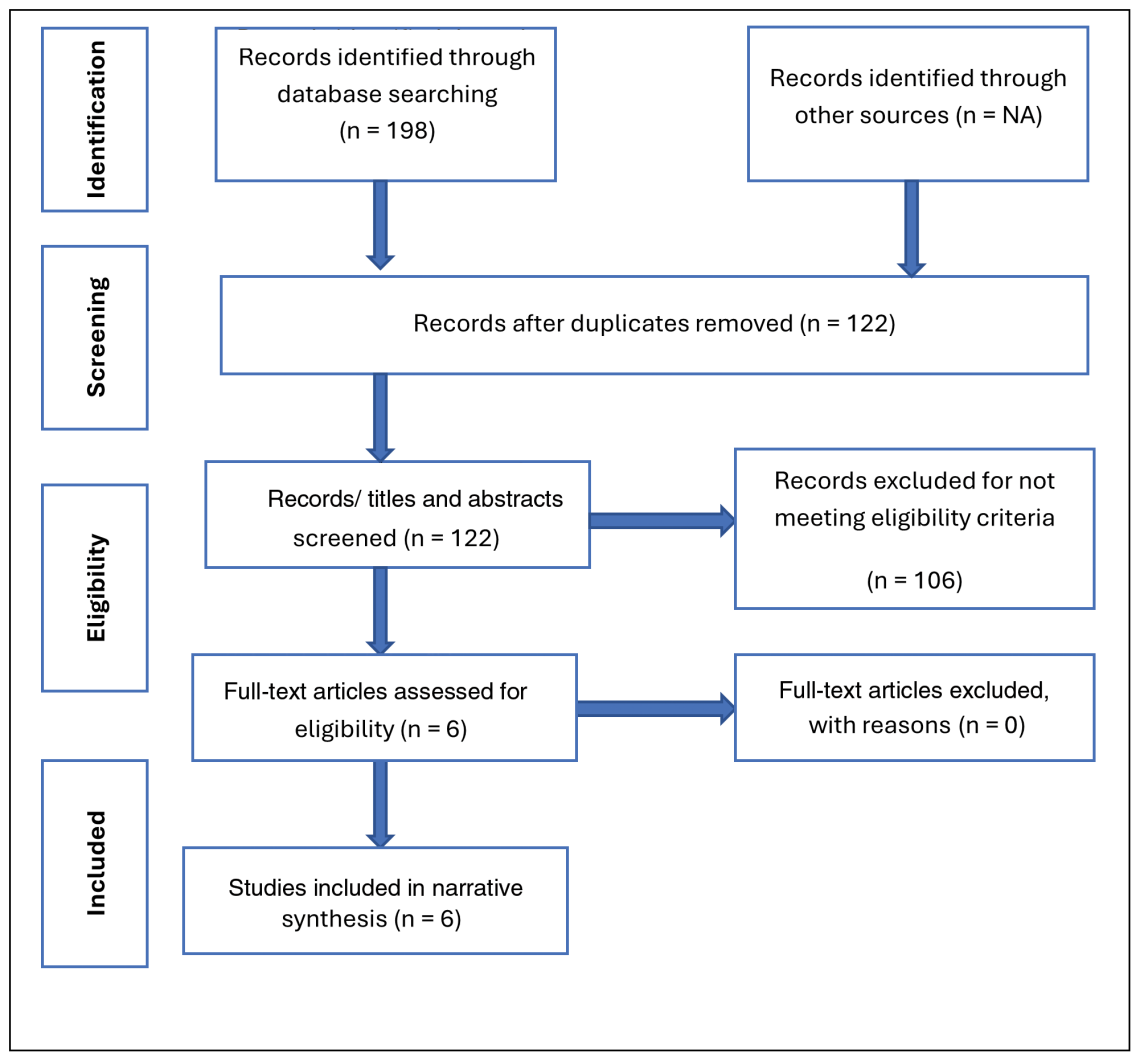
PRISMA Flow Diagram for the scoping review.

Study sampling and screening process resulted into a creation of a separate Endnote file was for each database (CINAHL=43, MEDLINE=113, and PsycINFO =42). The studies (n=198) were then collated and after removing duplicates, a total of 122 studies were retained and reported in the PRISMA Flow Diagram ( Figure 1, Table 2) and checklist for Scoping Reviews (PRISMA-ScR): https://doi.org/10.5281/zenodo.15550840.

**Table 2.** Scoping Review Articles.

Author	Year	Country	Title
Mkenda *et al.*	2016	Nigeria & Tanzania	Cognitive stimulation therapy as a low-resource intervention for dementia in sub-Saharan Africa (CST-SSA): Adaptation for rural Tanzania and Nigeria
Paddick *et al.*	2017	Nigeria & Tanzania	Cognitive stimulation therapy as a sustainable intervention for dementia in sub-Saharan Africa: feasibility and clinical efﬁcacy using a stepped-wedge design
Olakehinde *et al.*	2019	Nigeria	Managing dementia in rural Nigeria: feasibility of cognitive stimulation therapy and exploration of clinical improvements
Spector *et al.*	2019	Tanzania	Mixed methods implementation research of cognitive stimulation therapy (CST) for dementia in low and middle-income countries: study protocol for Brazil, India, and Tanzania (CST-International)
Stoner *et al.*	2020	Tanzania	A New Approach for Developing “Implementation Plans” for Cognitive Stimulation Therapy (CST) in Low and Middle-Income Countries: Results From the CST- International Study
Morrish *et al.*	2021	Tanzania	Group experiences of cognitive stimulation therapy (CST) in Tanzania: a qualitative study

Titles and abstracts were screened for inclusion. Two articles were excluded at this stage ^58, 59^. Full texts for remaining studies were then reviewed. The articles were reviewed by 2 independent reviewers. Decisions on inclusion were made independently and uncertainties were discussed by the authors.

### Data charting process and data items

Data charting process included weekly meetings were held to discuss and review the data charting form captured in Microsoft Excel ^57^. Table 3 shows the data extraction template.

**Table 3.** Data Extraction Template.

Study characteristics	Responses
Author	
Year	
Country	
Title	
Aim	
Design (e.g Qual/Quant)	
Method (e.g Interviews/focus groups/RCT)	
Participants (Number of people and who they were. Eg. patients or staff)	
Measures (QoL, Memory etc) and outcomes e.g. feasibility	
Results/Findings (Key findings)	

The data items or variables extracted included the following: author(s); year of publication; study location (country); title; aims of the study; study design; study methodology; study populations or participants; outcome measure; and key results and recommendations ^57^.

Table 4 presents the description of the studies. No quality assessment was undertaken ^57^. Thematic data analysis and synthesis followed the data charting stage to ensure further processing and categorisation ^57^. We identified the key themes emerging from the results sections of the qualitative articles ^6^. Results were then collated, summarized, and reported following the reporting standards for scoping reviews ^60^.

**Table 4.** Description of the Studies.

Author	Year	Country	Design	Methods	Sample size	Participants	Outcomes (Measures)
Mkenda *et al.*	2016	Nigeria & Tanzania	Qualitative	Formative method for adapting psychotherapy. Workshops and discussions in the UK. Feasibility assessments	n=21	People with dementia, carers, community health workers, village health workers, village leaders	Feasibility, acceptability, and adaptability
Paddick *et al.*	2017	Nigeria & Tanzania	Quantitative	Interventional study with stepped-wedge design. Randomization. Baseline, Pre-CST assessment, and post-CST assessment Interviews	n=34	People with dementia age 65 years and over and carers (no specifics for n for two groups)	Feasibility of CST. Costs of CST intervention. Quality of life (WHOQOL-Bref). Cognition (Alzheimer’s Disease Assessment Scale – Cognitive (ADAS-Cog), Anxiety and Depression (Hospital Anxiety and Depression Scale (HAD). Burden of care (Zarit Burden Inventory).
Olakehinde *et al.*	2019	Nigeria	Quantitative but no control group.	Interviews/Assessments	n=9	People with dementia (age 65 years), sample size (n=9, 5 males and 4 females).	Feasibility. Quality of life (WHOQOL-Bref). Cognition (ADAS-Cog). Activities of Daily Living. Neuropsychiatric behavior (Neuropsychiatric Inventory). Caregiver burden (ZBI). Disability (WHO Disability Assessment Schedule)
Spector *et al.*	2019	Tanzania	Mixed Methods Implementation Research Protocol	Mixed methods implementation research with four phases: (1) Exploration of barriers to implementation (2) Implementation plans (3) Evaluation in each country (4) Refinement & dissemination	Suggested n=50	Stakeholders’ meetings with clinicians, policymakers, people with dementia, and their families. Suggested sample (n=50) for each country.	CST adherence, attendance, acceptability, and attrition. Cognition (ADAS-Cog). QOL (WHOQOL-Bref). ADLs (Easy-Care Independence Scale) ^64^. Burden (ZBI and Dementia Caregiver Experience Scale (DemCarES)). Cost/affordability (Client Services Receipt Inventory and Resource Utilization in Dementia. Barriers and facilitators of CST implementation
Stoner *et al.*	2020	Tanzania	Consolidated Framework for Implementation Research (CFIR)	Three-stage mixed methodology. Methods consisted of 1) exploration of barriers to and facilitator of CST 2) development of implementation activities 3) development and monitoring of formal implementation plans	Tanzania n=49 stakeholders	Stakeholders are grouped in Tanzania into three: decision makers or policy professionals (n=5), healthcare professionals expected to deliver CST (n=33), and people to receive CST and their carers (n=11)	Barriers of CST, Facilitators of CST, Implementation activities
Morrish *et al.*	2022	Tanzania	Qualitative	Semi-structured Interviews	n=16 people with dementia n= 4 CST group facilitators	People with dementia age 70 years and over and group facilitators – occupational therapists	Experiences of being in a group. CST principles. Cognition. Quality of life

**QOL=quality of life, WHO=World Health Organization, ADAS-Cog=Alzheimer’s Disease Assessment Scale, WHODAS= WHO Disability Assessment Schedule**

## Results

### Selection of sources of evidence

Following the screening process/eligibility criteria, six articles were included in the scoping review ( Table 4, Figure 1). The eligibility criteria were guided by the PCC framework as described earlier ^43^.

### Description and characteristics of the selected studies

Table 4 presents the characteristics of the included studies. The six included studies covered only two African countries: Nigeria and Tanzania ^10, 29, 37, 38, 40– 42^. The CST research has been done by two teams based in West Africa (Nigeria) and East Africa (Tanzania) conducted the studies. Two studies covered both Nigeria and Tanzania ^37, 40^. One study covered Nigeria only ^41^ and three studies covered Tanzania ^29, 38, 42^.

The designs of the included studies varied. Two studies were quantitative. One was a randomized study with a stepped-wedge design ^40^. One study had no control group ^41^. Two studies were qualitative ^37, 42^. One study was a study protocol yet to be implemented ^29^.

Most studies covered people with dementia only ^10, 37, 40, 41^ while others targeted caregivers, community health workers, village health workers, and village leaders. Other studies focused on key stakeholders including policy makers, researchers, carers, and people with dementia ^10, 29, 37, 38, 40– 42^.

The studies recruited older participants (age 65 years and older) and their sample sizes varied from 9 ^41^ to 50 participants ^29^. One study reported a sample size of 16 participants aged 70 years and older and 4 group facilitators ^42^. One study did not report sample size ^37^. Another study reported a sample size of 34 but did not specify how many participants were people with dementia and how many were carers ^40^.

### Primary outcomes of CST in Africa

Table 4 shows the key CST outcomes and their measures or assessment tools. In the 6 articles, the following outcomes were reported: feasibility ^10, 37, 40– 42^, adaptability, acceptability, barriers, and facilitators of CST implementation and clinical outcomes ^29, 38, 40^. The most reported clinical outcomes were cognition and quality of life ^10, 40– 42^. Others were caregiver burden, activities of daily living (ADLs), disability, anxiety, and depression ^10, 37, 40– 42^. Clinical outcomes were reported in three studies ^37, 40, 41^. These are reported as follows:

First, cognition improved because of CST. The first trial of CST in SSA reported cognitive improvement post-CST intervention regarding new learning and memory. The ADAS-Cog mean scores changed from 15.5 to 11.4 ^40^. A Nigerian study reported that cognition improved (ADAS-Cog from 32.0 to 22.7) during pre-CST and post-CST respectively ^41^. A systematic review found evidence for improving cognition due to CST ^10^. A qualitative study in Tanzania reported mental stimulation after group activities and improvement in cognition and memory but did not report statistical outcomes ^37, 42^.

The CST intervention improved the quality of life of PWD ^10, 37, 40, 41^. A Nigerian study reported an improvement in quality of life of 89% as measured by WHOQOL-Bref ^41^. Several systematic reviews reported a strong association between CST and the quality of life ^6, 7, 52^. A systematic review similarly reported improvement in the quality of life as well ^10^. However, some RCTs found no significant association between CST and quality of life in Portugal ^61^.

There was a reduction in behavioral and psychological symptoms of dementia and caregiver burden after completion of the course ^40^. In addition, significant improvements in activities of daily living (ADL) were reported. For example, in one Nigerian study, the Zarit Burden Inventory (ZBI) ^62^ score decreased (suggesting improvement) from 18 to 11 ^41^. In Tanzania, there was an improvement in the neuropsychiatric symptom burden, number, and severity as measured by the neuropsychiatric inventory ^40, 63^.

## Discussion

### Adaptations and modifications of CST to Africa

CST was adaptable to cultures in Africa ^37, 40– 42^. The group sessions are highly relevant to the communal lifestyle and extended family systems in many African settings. Cultural adaptation is a key element of the CST ^37, 38^. The process included translation into local languages ^29, 37, 38^.

CST was first adapted for use in Africa - CST-SSA, in 2016 ^37^, following a recognized method of adapting psychotherapeutic interventions ^25^. The CST-SSA was piloted in Tanzania and Nigeria ^29, 40– 42^, which resulted in recommendations for further refinement and modification ^37^. The initial modification of CST, CST-SSA maintained the 14 sessions of the original CST ^26, 37^. Recommended changes included the identification of suitable treatment settings, task adaptation to accommodate illiteracy, awareness of cultural differences, and use of locally available materials and equipment to ensure sustainability ^37^. Further adjustments included the use of local current affairs and village news in the fifth (current affairs) session, local maps instead of national maps for the tenth (orientation) session, and using local materials and equipment for the task adaptation session ^29^.

In the published protocol for CST-International ^29^, the number of sessions is maintained - 14 sessions over 7 weeks using a manual. For task adaptation, the use of local materials and equipment is recommended. Selecting a meeting place acceptable to all is recommended ^38^. Places of worship are recommended to be avoided as these may cause acceptability challenges for those from other denominations or religions ^37^. Conducting two sessions on the same day is strongly recommended to reduce travel time. However, the protocol for CST-International has not yet been implemented and evaluated ^29^.

### Feasibility and acceptability of CST in Africa

The key assessment of feasibility in the CST studies was the overall attendance rate. The CST-SSA was highly feasible in African settings ^37, 40, 41^. High attendance rates (81%) were recorded for CST ^65^. Attrition resulted from expecting medication intervention ^37, 40, 41^. 

CST was highly acceptable by the population including participants and caregivers in Nigeria and Tanzania ^10, 37, 40, 41^. CST intervention was acceptable to both participants and caregivers. The group sessions appeared very therapeutic for older people ^41^. In one Nigerian study, no one dropped out of the entire course - 11 sessions had full attendance, and three sessions had eight people in attendance (98%) ^41^. In the Hai district of Tanzania, 5 participants completed the program and two dropped out ^37^. Attrition rates were higher in Nigeria than in Tanzania. To increase acceptability, selecting a meeting place acceptable to all helps ^29, 37^.

### CST content, delivery and implementation

Group sessions were a key aspect of the CST delivery. Groups are recommended to be composed of between 5–8 people ^10, 37, 38, 40^. People with dementia are encouraged to reflect, concentrate, and engage their memories through the practical and outdoor activities held in each session. These should take place in a relaxed environment ^37, 38, 42^. This is beneficial for the African context.

In addition, CST-SSA can be delivered by non-specialist staff, including social workers and not necessarily medical personnel ^41^. The facilitators guide the group sessions. They can be specialist staff such as nurses, doctors, and occupational therapists who are trained to facilitate the group sessions. However, they can also be non-specialized personnel ^29, 37, 38^.

The implementation cost of the CST intervention, assuming 14 sessions per intervention and eight participants per group was 268 USD per session. The 14 sessions were conducted over seven weeks with two sessions per week ^29, 37^. The CST manuals were used ^6^. The cost per participant per complete course was 34 USD. The mean costs or expenditure for formal healthcare by the patients was low – about $1.18 ^40^. Even though it appears affordable, most people would not afford it without financial support.

Several costs for implementing CST were reported. These include transportation reimbursement ($2) to motivate participants to return for sessions and giving a small gift to older people to take to their grandchildren ^37^.

### Barriers of CST implementation in Africa

Group conflicts were reported as a barrier to CST implementation ^37, 38, 40, 42^. Group conflicts arose from previous challenges over land ownership; fears of gossip; tribal and religious differences in Tanzania ^42^; selection of venues for the meeting in places of faith ^40^ and oversharing of personal information ^37, 38^.

A shortage of qualified professionals and specialized healthcare workers (such as occupational therapists, nurses, and doctors) was viewed as a key barrier. CST was viewed as additional work for both healthcare professionals and caregivers ^37, 38^.

Facilitator-provider language barrier was noted ^42^. The lack of awareness about dementia among the participants and their caregivers and the lack of understanding about psychosocial interventions including CST among people with dementia were further barriers ^37, 41^.

Low literacy attainment among the participants affected some sessions. The orientation session could not use maps since many participants were unable to read and write ^38^. Tasks involving holding a pen and reading words were not appropriate for older persons not accustomed to writing ^37^.

Poor transportation and infrastructural challenges limited travel to group sessions ^37, 38, 40^. Seasonal roads and rainy seasons complicated travel to venues for meetings. Transportation difficulties increased travel time and inflated travel costs ^37^. Missing sessions were due to transport and logistical challenges, illness, and family events ^40^.

Resource constraints and logistical challenges limited the sessions’ smooth running ^37, 38^. Some sessions needed equipment that required electricity, which was unavailable. The privacy of location settings was critical but was difficult to achieve in some settings ^38^.

Cultural considerations limited CST’s impact as older people are expected to be relieved of household chores and duties ^37^. This contradicts CST principles of encouragement of mentally stimulating activities. Another cultural issue identified in Tanzania was the confusion over the names of participants ^37^.

Lifestyle and work arrangements posed challenges for CST in rural areas that prioritized village events such as burials and weddings ^37^. Some older people tended to miss sessions in favor of these events. Contingency plans were required for those who missed sessions ^10, 37^.

There was a general preference for tangible medical treatment instead of psychological interventions ^10^. Morbidity due to non-communicable diseases in old age made offering non-pharmacological interventions difficult as some participants expected medical treatment for their health conditions ^37^. The critical issue surrounding multimorbidity and the unmet need for healthcare among older persons and their preference for pharmacological treatment is cross-cutting in most studies ^37, 40– 42^. A CST study in Brazil also reported poor motivation for CST due to expectations in medical treatment ^66^. Therefore, for CST to be successful, managing such expectations is flagged as a critical component of the intervention. Finally, visual impairments without eye care among some older people limited their engagement with CST. Therefore, screening for CST should include a brief eye exam ^38^.

### Facilitators of CST in Africa

Several facilitators for CST-Africa were identified. First, caregiver engagement motivated participation ^29^. Engaging caregivers reduced their boredom and the psychological anxiety of waiting during sessions.

Second, group sessions were highly motivating for older persons. Being around others distracted many participants from physical illness ^10, 40, 42^. Many participants were able to work together and remind each other of good memories ^42^. Third, medical check-ups for blood pressure and appropriate referrals motivated participant engagement in both Nigeria and Tanzania ^37, 42^. Fourth, CST-SSA can be delivered by trained non-medical personnel ^41, 42^. Fifth, positive group experiences such as shared memories, group cohesion, and personal development motivate many older persons with dementia ^42^. Participants often looked forward to attending the next group sessions due to the created sense of belonging ^42^. Finally, the provision of transportation and refreshments was an important incentive for participation in the CST group sessions ^42^.

### Future directions in dementia care in Africa

While our study demonstrates that culturally adapted CST is feasible, acceptable, and potentially beneficial for people with dementia in African settings, these findings also highlight an urgent need for broader healthcare system integration and supportive public policies to sustainably improve dementia care throughout sub-Saharan Africa ^67, 68^. Dementia is increasingly recognized as a major public health priority in Africa, yet most countries currently lack national dementia strategies or policies to guide dementia diagnosis and care by healthcare providers and caregiver support systems. The World Health Organization’s Global Action Plan on Dementia (2017–2025) provides a framework for Member States to develop or update dementia policies, enhance risk reduction and diagnosis, and implement caregiver support and awareness initiatives, but few national governments have implemented policies in line with this plan ^69^.

Strengthening public policy could include covering cognitive health and dementia care within universal health coverage and primary healthcare platforms, training community health workers in identifying symptoms of dementia, and providing support to caregivers of people with dementia in their communities. Moreover, the addition of culturally-adapted psychosocial interventions like CST are critical to include within the national policies on dementia care that protect cognitive health and support caregivers in sub-Saharan Africa.

### Strengths and limitations

The strengths of this study are as follows: First, this is the first scoping review to be conducted specifically about CST for older persons with dementia in Africa. Second, methodologically there was consensus from two researchers which increased the reliability of data charting ^57^. Third, some studies reported adequate sample sizes which provided rich data ^42^. Fourth, this scoping review has provided a comprehensive overview of CST-SSA implementation. Finally, the review used a systematic and reproducible search strategy using a scoping review framework ^57^.

However, several limitations merit discussion. We limited this review to articles published in English hence excluding Francophone Africa ^53^. Some studies report a limitation in asking people with dementia to detail experiences in the past, as recall is likely to be limited ^42^. Translation to address language barriers could have introduced some nuances of translation and interpretation ^42^. Due to the limits of time, we were unable to include grey literature.

### Recommendations

Our recommendations consider CST implementation in African nations, future practice, and research.

### CST implementation in Africa

CST is important in Africa over other options for therapy that may be available (pharmacological treatment, other treatments) because it is cost-effective and can be delivered by trained non-medical personnel. Group therapy or sessions are relevant to the extended family system in Africa.

Having synthesized the findings of all six papers we suggest that the intervention is delivered in two sessions on the same day each week to reduce travel time for older persons and their carers ^29, 37^. In addition, contingency plans to reschedule sessions missed due to village events would be beneficial ^37^. Delivering the intervention on village market days should be avoided wherever possible. Dementia awareness courses for family carers should highlight the stigma associated with dementia ^38^. Selecting a meeting place acceptable and neutral to all, and avoiding places of worship, such as village offices or halls is important ^29, 37^, as is support from village committees is critical for success ^37^. Refreshments for group sessions are key for older people ^29, 37, 38^. Also, it is good to give participants a small gift, such as a confectionary, to give to a grandchild, following attendance ^37^. To deal with expectations of medical treatment and to be ethical, blood pressure screening and appropriate referral by nursing staff are important ^37, 42^.

However, significant barriers to its implementation of CST on a large-scale across the African continent need to be highlighted. First, a pathway to train personnel employed within individual hospitals or on the community level are needed. Next, a referral pathway for clinicians must be created in order for clinicians suspecting dementia in their patients to refer to a trained personnel who delivers CST. Moreover, CST may be best delivered at the community level, particularly in rural settings that may be distanced from a healthcare facility. This will take the collaboration and the support of the Ministries of Health of each individual country. With support from international organizations focused on Alzheimer’s dementia diagnosis and prevention, there may be the possibility to create research networks on using CST throughout Africa in order to better determine the feasibility, acceptability and outcomes associated with CST application in the African context. 

### Implications for practice

In the future, CST implementation in African nations should include groups of the same tribe or religion, achieve a gender balance, and provide refreshments ^42^. Group facilitators are to manage medical expectations on the part of the participants ^37^.

Training local people as facilitators who understand cultural dialects could improve the acceptance of the CST ^42^.

### Implications for future research

Future research should include high-quality studies of the effectiveness of CST in African countries to test its effectiveness in the improvement of cognition and quality of life. To date, only one RCT of CST has been conducted, in Tanzania ^40^. Developing research proposals to pilot the CST-SSA is warranted ^41^.

## Conclusions

CST is feasible and has been adapted to African contexts and piloted in two African countries (Nigeria and Tanzania). Although there are some barriers to overcome, CST has the potential to make a significant impact, by improving the quality of life and reducing the burden for the carers of older persons living with dementia in Africa. Finally, CST improves clinical outcomes including cognition and quality of life among older persons.

The next steps include implementing a study to estimate the prevalence of dementia among older persons in Uganda (2023–2026). This is needed as preliminary data for piloting and implementing the CST intervention study among older people with dementia in Uganda (2028–2032).

## Informed consent statement

Not Applicable

## Ethics approval and consent to participate

The study is based on secondary data. Ethical approval was not required.

## Declarations

### Consent for publication

Not Applicable

## Data Availability

Zenodo. Endnote Library for Cognitive Stimulation Therapy among older persons with dementia in Africa: A Scoping Review. https://doi.org/10.5281/zenodo.7957124 ^70^. This project contains the following underlying data: 3_CST SR_Duplicates Removed_04022021_Exclusion Criteria Applied_09022021.Data (Data which supports the opening of the Endnote file. Has some few PDF files for some selected articles). 3_CST SR_Duplicates Removed_04022021_Exclusion Criteria Applied_09022021.enl (Endnote Data File with Articles selected for the review and their abstracts). Sbd (Endnote System File). Data are available under the terms of the Creative Commons Attribution 4.0 International license (CC-BY 4.0). This project contains the following extended data: PRISMA checklist and Supplementary Table 1. Search strategy. Zenodo: PRISMA ScR checklist and flow chart for ‘Cognitive stimulation therapy for older people with dementia in Africa: A scoping review’ ^71^. https://doi.org/10.5281/zenodo.15550840. Zenodo: Supplementary Table 1: Search Strategy. https://doi.org/10.5281/zenodo.15549753 ^54^. Data are available under the terms of the Creative Commons Attribution 4.0 International license (CC-BY 4.0).
