# Supplementary material for: Cognitive Stimulation Therapy for older people with Dementia in Africa: A Scoping Review
Source: Open Res Afr. 2026 Jan 16;6:10. Originally published 2023 Jun 13. [Version 4] doi: 10.12688/openresafrica.14092.4 (PMC12859423; doi:10.12688/openresafrica.14092.4)
Supplement: Peer review history

# Reviewer report: Reviewer response for version 4

**DOI:** 10.21956/openresafrica.17594.r33582

**Published:** 2026-01-30

**Authors:** Federica Piras

**Affiliations:** 1 IRCCS Fondazione Santa Lucia, Rome, Italy

I think that the paper was slightly improved as for example, results are more clearly delineated in the present version. Considering that dementia diagnosis and care strategies are scarcely implemented within the target geographic area, my opinion is that it would be important to disseminate the paper as a first step toward a systematic approach. I indeed very much appreciated the section "Future directions." and the recommendation given for a broader CST implementation in SSA.  Although below the European standards for a scientific indexing, I think the paper can be indexed (but please, correct some misspellings).

Are the rationale for, and objectives of, the Systematic Review clearly stated?

No

Is the statistical analysis and its interpretation appropriate?

Not applicable

Are sufficient details of the methods and analysis provided to allow replication by others?

Partly

Are the conclusions drawn adequately supported by the results presented in the review?

Partly

Reviewer Expertise:

I authored several papers on the Italian CST adaptation and its efficacy in dementia patients from different etiologies. My research interests span from neuropsychology, to cognitive remediation and stimulation in different neurodegenerative and neuropsychiatric diseases.

I confirm that I have read this submission and believe that I have an appropriate level of expertise to confirm that it is of an acceptable scientific standard.

# Reviewer report: Reviewer response for version 3

**DOI:** 10.21956/openresafrica.16980.r32313

**Published:** 2025-11-06

**Authors:** Federica Piras

**Affiliations:** 1 IRCCS Fondazione Santa Lucia, Rome, Italy

I see that some changes have been made, although authors have not replied to my comments. Specifically, the Rationale and Study objectives seem to be more congruent now, although I still suggest to better define why the primary outcomes of the intervention are also discussed (unless they want to highlight potential differences in outcomes, depending on slight modifications of the original protocol). In the discussion section, I would like to read something more detailed on the current public policy and strategies to fill the present gaps regarding dementia care in Africa. For example, I came across a quite interesting paper by Akinyemi and the African Dementia consortium [Reference 1] that authors may cite and discuss.

Are the rationale for, and objectives of, the Systematic Review clearly stated?

No

Is the statistical analysis and its interpretation appropriate?

Not applicable

Are sufficient details of the methods and analysis provided to allow replication by others?

Partly

Are the conclusions drawn adequately supported by the results presented in the review?

Partly

Reviewer Expertise:

I authored several papers on the Italian CST adaptation and its efficacy in dementia patients from different etiologies. My research interests span from neuropsychology, to cognitive remediation and stimulation in different neurodegenerative and neuropsychiatric diseases.

I confirm that I have read this submission and believe that I have an appropriate level of expertise to confirm that it is of an acceptable scientific standard, however I have significant reservations, as outlined above.

## References

[1] : Dementia in Africa: Current evidence, knowledge gaps, and future directions. *Alzheimer's & Dementia* .2022;18(4) : 10.1002/alz.12432 790-809 10.1002/alz.12432 PMC895762634569714

# Reviewer report: Reviewer response for version 2

**DOI:** 10.21956/openresafrica.16794.r32022

**Published:** 2025-03-27

**Authors:** Maria Vânia Silva Nunes

**Affiliations:** 1 Universidade Católica Portuguesa, Lisbon, Portugal

I appreciate the opportunity to review this article and commend the authors for choosing such a relevant topic. However, in methodological terms, I believe there are some important aspects to highlight.

 Regarding the abstract, it is not sufficiently clear about the methodology or the results. Some aspects of the line of reasoning in the introduction are also unclear. Most of it focuses on CST, with little being mentioned, in terms of the review, about what justifies looking at its application in Africa.

 When moving to the rationale and methods, having chosen a PCC, it was also not clear to me what emerges in terms of the main question. Outcomes are also mentioned, which do not usually form part of the PCC, but these are not integrated into the search (e.g., quality of life, feasibility, etc.). It seems to me that the definition should be more restrictive, which would make the objectives of the review clearer.

 Being a scoping review, the search was quite limited in terms of sources, which restricts the main objective, which would be mapping. Regarding the searches, the terms are mentioned without considering MeSH. The strategy, which should be presented in full for at least one database is not. There are some minor inconsistencies between the text and what appears in the PRISMA flowchart, as well as between the flowchart and the number of included articles.

 The tables also do not follow what is usual in this type of work, resulting in duplications. Since it was not entirely clear what was being sought, interpreting the data was also not straightforward for me. This is particularly evident in the outcome aspect, which considers elements at completely different levels.

 The fact that none of the tables identify the articles that are later discussed makes reading difficult, forcing constant reference to the bibliography.

 Overall, despite the relevance and the evident work involved, unfortunately I believe there are aspects that diminish the importance of indexing this work in its current state.

Are the rationale for, and objectives of, the Systematic Review clearly stated?

No

Is the statistical analysis and its interpretation appropriate?

Not applicable

Are sufficient details of the methods and analysis provided to allow replication by others?

Partly

Are the conclusions drawn adequately supported by the results presented in the review?

Partly

Reviewer Expertise:

Dementia, Neuropsychology

I confirm that I have read this submission and believe that I have an appropriate level of expertise to state that I do not consider it to be of an acceptable scientific standard, for reasons outlined above.

# Reviewer report: Reviewer response for version 2

**DOI:** 10.21956/openresafrica.16794.r31917

**Published:** 2025-01-13

**Authors:** Daniel Mograbi

**Affiliations:** 1 Pontifical Catholic University of Rio de Janeiro, Rio de Janeiro, State of Rio de Janeiro, Brazil

The authors did very few of the suggested revisions, I’m afraid I have to recommend 'Not Approved'.

Are the rationale for, and objectives of, the Systematic Review clearly stated?

Partly

Is the statistical analysis and its interpretation appropriate?

Not applicable

Are sufficient details of the methods and analysis provided to allow replication by others?

Partly

Are the conclusions drawn adequately supported by the results presented in the review?

Partly

Reviewer Expertise:

Cognitive neuroscience/experimental neuropsychology

I confirm that I have read this submission and believe that I have an appropriate level of expertise to state that I do not consider it to be of an acceptable scientific standard, for reasons outlined above.

# Response

**Published:** 2025-02-28

**Authors:** Stephen Ojiambo Wandera

**Affiliations:** Population Studies, Makerere University, Kampala, Central Region, Uganda

Dear Reviewer,

 Thank you for the comments. We addressed the comments. It seems the format used is challenging for us - not easy to show track changes. This is something the journal can address in future. Kindly share the key comments you need us to address.  We are willing to address them again.

 Thank you

# Reviewer report: Reviewer response for version 2

**DOI:** 10.21956/openresafrica.16794.r31955

**Published:** 2025-01-08

**Authors:** Federica Piras

**Affiliations:** 1 IRCCS Fondazione Santa Lucia, Rome, Italy

**Dear authors,**

 I carefully considered the scoping review you authored as I think that more studies are needed to evaluate the feasibility and adaptability of the CST protocol across various cultures, in contrast to the context for which it was originally designed. Nevertheless, while it is indeed deserving of consideration, I personally think that the article present form lacks the requisite maturity for indexing, particularly due to its failure to fully achieve its intended objectives.

**Please, find below some suggestions to (hopefully) improve the paper focus:**

**Abstract:**

- Years reported for the search in the Methods section (2000-2021) differ from what is stated in the manuscript (2003-2021).
- At the end of the results section the sentence “ *Although there are some barriers to overcome, CST has significant facilitators in an African context*.” it’s not entirely clear to me.

**Introduction:**

- Apart from describing the incidence, causes and consequences of dementia (two paragraphs that should be more concise since do not really convey new information) it would be crucial to add more specifics regarding the actual public policies and available services to manage dementia in Africa. I understand that it might be very difficult given the vastity of the African continent, but since the intervention was adapted for the country in 2016 (or was the adaptation only for sub-Saharan Africa?) my guess is that Mkenda *et al*., or even Stoner *et al*., 2020 accurately surveyed the resources available before providing their CST adaptation plans for rural or low/middle-income countries.
- Indeed, from the last paragraph of the introduction it seems that you intended to evaluate the program adaptability, feasibility and efficacy only as a potential alternative to unavailable (in rural Africa) dementia medications. However, the program was originally designed to overcome the medicalization of dementia, and recent studies demonstrated that it's equally (or even more) effective than current available drugs (Woods *et al*., 2023 ^1^). Since CST has demonstrated efficacy in postponing the cognitive decline usually expected in mild-to-moderate dementia, the program is positioned to become a global standard of care. I would suggest to focus the introduction section on the solid support for the increased adoption of CST as a non-pharmacological intervention for dementia on a global scale, thereby situating your manuscript within a wider international discourse.
- To me the Rationale and Study objectives sections are not entirely congruent. Actually, in the Rationale you state that CST efficacy has already been solidly established, while its applicability to the African context (pondering the potential barriers and facilitators and the possible adaptations needed to adjust specific cultural needs), the feasibility (I assume considering the already available resources) and the acceptability of the program are yet to be determined. In the following paragraph you anticipate that information regarding these issues was derived from published literature, although the listed factors (“ *identify the types of available evidence, clarify key concepts/ definitions in the literature, describe the primary outcomes of CST; examine how research is conducted on a CST, identify key characteristics or factors related to CST, and identify and analyze gaps in the CST knowledge base*”) seem not to be related to the declared aims. Then in the Study objectives you enucleate the information that you intended to extract (study design -probably to clarify how the program has been thus far implemented in the African context-, adaptations/modifications, barriers and facilitators) although you also considered primary outcomes without clarifying how this might help in understanding the issues you projected to debate. I think that both sections need a thorough revision in order to clarify the readers the real objectives of your work.

**Methods:**

- The subparagraph Study design is a mere repetition of the following Eligibility criteria and should be removed. You should also clarify (see above) whether you extracted only information regarding CST feasibility, adaptability, implementation, and barriers/facilitators within the sub-Saharan Africa context (in accordance with the stated rationale) or if the potential program outcomes were also considered (did you intend to verify whether a specific implementation/adaptation determined different results? Please, clarify). Exclusion criteria are quite confusing (I doubt your search resulted in animal/children studies being included); plus, you said that protocols were excluded, but then in the Results section (Description and characteristics of the studies) a yet to be implemented study protocol is included.
- To me Table 2 is not necessary. Please, include the search string and list the search terms for Africa elsewhere.
- In the PRISMA flow diagram 7 studies are reported as included (while only 6 are presented and discussed).

**Results:**

- Description and characteristics of the studies: again, 7 studies are included. Please, clarify whether in studies including larger sample sizes (34 or 50) several consecutive interventions were conducted (given that CST is usually performed in groups including a maximum of 8 participants).

**Discussion:**

- The considered factors (adaptability, feasibility, acceptability, barriers/facilitators and implementation) should be reported in the results section. Again, I suggest not to include the program outcome as this matter is not subject to debate (unless it is framed within a theoretical context, see above).
- I expect to read in the amended version a proper discussion on the to-be-considered factors to hopefully deliver the CST program on a larger scale within the African context (something more than the very brief subparagraph “CST implementation in Africa”). Indeed, barriers to implementing evidence-based interventions can occur at multiple levels of a health system and I find the absence of an exhaustive representation of the economic, cultural, organizational, and policy challenges that are currently hindering a wider dissemination of the program in the African continent.

Are the rationale for, and objectives of, the Systematic Review clearly stated?

No

Is the statistical analysis and its interpretation appropriate?

Not applicable

Are sufficient details of the methods and analysis provided to allow replication by others?

Partly

Are the conclusions drawn adequately supported by the results presented in the review?

Partly

Reviewer Expertise:

I authored several papers on the Italian CST adaptation and its efficacy in dementia patients from different etiologies. My research interests span from neuropsychology, to cognitive remediation and stimulation in different neurodegenerative and neuropsychiatric diseases.

I confirm that I have read this submission and believe that I have an appropriate level of expertise to state that I do not consider it to be of an acceptable scientific standard, for reasons outlined above.

## References

[1] : Cognitive stimulation to improve cognitive functioning in people with dementia. *Cochrane Database of Systematic Reviews* .2023;2023(1) : 10.1002/14651858.CD005562.pub3 10.1002/14651858.CD005562.pub3 PMC989143039804128

# Reviewer report: Reviewer response for version 2

**DOI:** 10.21956/openresafrica.16794.r31918

**Published:** 2025-01-03

**Authors:** Diana Karamacoska

**Affiliations:** 1 NICM Health Research Institute, Western Sydney University, Penrith, Australia

I’ve reviewed the revised paper and wish to submit my approval of the indexing. I have no further comments.

Are the rationale for, and objectives of, the Systematic Review clearly stated?

Yes

Is the statistical analysis and its interpretation appropriate?

Partly

Are sufficient details of the methods and analysis provided to allow replication by others?

Partly

Are the conclusions drawn adequately supported by the results presented in the review?

Partly

Reviewer Expertise:

Dementia is my area of expertise and I have published several review articles, including integrative scoping and systematic reviews.

I confirm that I have read this submission and believe that I have an appropriate level of expertise to confirm that it is of an acceptable scientific standard.

# Reviewer report: Reviewer response for version 1

**DOI:** 10.21956/openresafrica.15249.r31339

**Published:** 2024-07-18

**Authors:** Daniel Mograbi

**Affiliations:** 1 Pontifical Catholic University of Rio de Janeiro, Rio de Janeiro, State of Rio de Janeiro, Brazil

Dear authors,

 I appreciate the opportunity to review the article. However, it requires several modifications before it can be accepted.

 Below are some suggestions for modifications, organized by topics and paragraphs.

 The authors are encouraged to consider consolidating similar themes into longer paragraphs throughout the study.

**Introduction:**

 To enhance clarity and coherence, ensure that sentences with similar themes are grouped together within paragraphs, i.e., demographics in the first and second paragraph; definition of CST in the fourth paragraph (fourth and tenth sentences). Additionally, consider consolidating short paragraphs, as there are currently several instances where paragraphs consist of only three to four sentences.

 Avoid repetition, i.e., Introduction, fourth paragraph, sentences regarding cost-effectiveness of CST.

 ‘ *(…) CST among older people with dementia and Africa*’ – replace ‘ *and*’ with ‘ *in*’.

  ‘(…) *research is conducted on a CST’* - remove ‘ *a*’.

**Methods**

*‘Design, protocols and registration/Eligibility’*

 The first study on CST was published in 2003, so why search for articles before this period?

 Avoid repetition of themes across different paragraphs: First, second and third paragraphs repeat information regarding population and intervention.

 ‘ *(…) , faces/senses’ – *the session is ‘faces/scenes’.

*‘The outcomes for the scoping review included: improvement in cognition, quality of life, and activities of daily living’ – *the review includes other outcomes.

 ‘ *Information source*’ and ‘ *Selection of sources of evidence*’ have repeating themes/sentences.

*Selection of sources of evidence*

 ‘ *Study sampling and screening process resulted into a creation of a separate Endnote file was*’ - exclude ‘ *was*’.

*Data charting process and data items*

 ‘ *Data charting process included weekly meetings were held to*’ - exclude ‘ *were*’.

 Table 5 – ‘ *Morrish et al., 2021’- *the year is 2022.

**Results**

*Description and characteristics of the studies*

 ‘ *The designs of the included studies varied. Two studies were quantitative. One was a randomized study with a stepped-wedge design, while the other had no control group. Two studies were qualitative. One study was a study protocol yet to be implemented. One study was a systematic review of psychosocial interventions for dementia in low- and middle-income countries. Another study was an implementation study.*’ – consider revising to enhance clarity and coherence.

 ‘ *Six studies took place in Tanzania while four out of six studies were in Nigeria*’ - the scooping review regards seven studies, rewrite this to clarify.

**Discussion**

 First paragraph mentioning India and Brazil - relate the findings in Brazil and India to those in Africa.

 ‘ *The adaptation process includes translation into local languages and the use of group facilitators*’– use of group facilitators to do what?

 ‘ *CST was first adapted for use in Africa (CST-SSA) in 2016*’ – so why covering papers from before this period?

*'The CST-SSA was piloted in Tanzania and Nigeria, which resulted in recommendations for further refinement and modification. There is now a published protocol for international use of CST (CST-International)’– *The connection between these two sentences are not clear.

 ‘ *There is now a published protocol for international use of CST (CST-International)*’ – the cited studies regard a study protocol for international implementation and not an international CST protocol.

 ‘ *Generally, the initial adaptation of CST, CST-SSA maintained the 14 sessions of the original*’ -Regarding ‘generally, I suggest using a different word here, as the current one doesn't make sense in this context.

 ‘ *Generally, the initial adaptation of CST, CST-SSA maintained the 14 sessions of the original CST. Recommended adaptations included the identification of suitable treatment settings, task adaptation to accommodate illiteracy, awareness of cultural differences, and use of locally available materials and equipment to ensure sustainability. Further adaptation included the use of local current affairs and village news in the fifth (current affairs) session, local maps instead of national maps for the tenth (orientation) session and using local materials and equipment for the task adaptation session.*’ – use the word ‘adaptation’ only once and replace others with synonyms to improve readability.

*‘In CST-International 23, the number of sessions is maintained - 14 sessions over 7 weeks using a manual. For task adaptation, the use of local materials and equipment is recommended. CST-International recommend selecting a meeting place acceptable to all. Places of worship are recommended to be avoided as these may cause acceptability challenges for those from other denominations or religions. Conducting two sessions on the same day is strongly recommended to reduce travel time. However, CST-International is still a protocol and its implementation has not yet been evaluated’ – *at present, there is no established international CST protocol – see above. However, this could be rephrased to mention the protocol suggested in the CST international study.

 ‘ *CST is highly adaptable for the African context and is highly acceptable by the population including participants and caregivers in Nigeria and Tanzania.*’ – this statement is repetitive, as part of it has already been mentioned earlier and it needs to be further developed.

*Feasibility of CST in Africa*

 Regarding this session, consider separating the presentation of the results from the discussion and recommendations about them.

*‘CST-SSA is highly feasible in African settings* *^25^* *^–^* *^27^* *. The 14 sessions* *^23^* *^,^* *^25^* *and CST manuals are very helpful* *^6^* *. High attendance rates (81%) were recorded* *^52^* *. Attrition resulted from expecting medication intervention* *^25^* *^–^* *^27^* *. However, the challenge is obtaining funding for such sessions*.’ - this paragraph could benefit from a revision to enhance clarity and coherence and suggestions should be presented in the recommendations/CST implementation in Africa.

 ‘ *Generally, the adapted CST-SSA was feasible in Africa*’ – this is repeating a statement present in the previous sentence.

 ‘ *Transportation reimbursement ($2) assured participants returned for sessions. Giving a small gift to older people to take to grandchildren was quite motivating25. CST-SSA can be delivered by non-specialist staff*’ -the sentences need to be combined cohesively.

*‘Missing sessions were due to memory deterioration, transport and logistical challenges’ – *transportation is a logistical challenge.

 ‘ *In one Nigerian study, no one dropped out of the entire course - 11 sessions had full attendance and three sessions had eight people in attendance (98%).*’ – provide reference.

 ‘ *In the Hai district of Tanzania, 5 participants completed the program and two dropped out while in Lalupon, Nigeria*.’ – this sentence starts by discussing one country and ends by discussing another.

*Content, delivery and implementation of CST in Africa*

 First paragraph: ‘(…) *helps with isolation of elderly people and isolation is a risk factor for dementia.’ – *provide reference.

*Outcomes of CST in Africa*

 ‘ *The most used outcomes were cognition and quality of life. Other outcomes were caregiver burden, and activities of daily living (ADLs). Other outcomes of the studies included disability measured by the WHO Disability Assessment Schedule (WHODAS), Neuropsychiatric Inventory (NPI), and Hospital Anxiety and Depression Scale (HADS). Several clinical outcomes were reported. There were only three studies that were quantitative and reported clinical outcomes*’ *. - *this sentence could be rewritten to improve readability and cohesion.

 ‘ *The first trial of CST in SSA reported cognitive improvement post CST intervention regarding new learning and memory (ADAS-Cog mean scores changed from 15.5 to 11.4*’ – provide reference.

 ‘ *The first trial of CST in SSA reported cognitive improvement post CST intervention* *(…).* *There was a reduction in behavioral and psychological symptoms of dementia (BPS) and caregiver burden after completion of the course. (…) Therefore, cognitive improvement is a key outcome of the CST.*’ – this paragraph combines results on cognition, but there is one sentence regarding bps and caregiver burden that seems out of context. This should be included in the later paragraph that discusses those outcomes.

 ‘ *Quality of life has been reported to improve with CST intervention* *(…)*’ - this paragraph could be rewritten to improve readability and cohesion.

*Barriers of CST implementation in Africa*

 ‘ *Some studies highlighted the barriers of CST implementation*’ – this first sentence should either include all the references cited in this paragraph or exclude them entirely, and references appear in each appropriate sentence.

 ‘ *These challenges include: Group conflicts arising from previous challenges over land ownership and fears of gossip. Other causes of group conflicts included tribal and religious differences in Tanzania.*’ – change ‘G’ in ‘Group’ to lower case, insert all the references and combine the sentences into one, separated by a semicolon, as they refer to the same topic.

 ‘ *CST was viewed as additional work for both healthcare professionals and caregivers.*’ – provide reference.

 ‘ *(…) Non-medical personnel can be trained to deliver the CST*’ – is this a barrier or facilitator?

 ‘ *Poor transportation and infrastructural challenges limited travel to group sessions* *’ - *this first sentence should either include all the references cited in this paragraph or exclude them entirely, and references appear in each appropriate sentence.

 ‘ *Resource constraints and logistical challenges limited the sessions’ smooth running’ *- this first sentence should either include all the references cited in this paragraph or exclude them entirely, and references appear in each appropriate sentence.

 ‘ *Cultural considerations limited CST’s impact as older people are expected to be relieved of household chores and duties. This contradicts CST principles of encouragement of mentally stimulating activities*.’ – provide references to each statement.

 ‘ *There was a general preference for tangible medical treatment instead of psychological interventions, the. rational*’ – remove ‘ *.*’ before ‘ *rational*’.

 ‘ *Finally, visual impairments without eye care among some older people limited their engagement with CST, screening for CST should include a brief eye exam*.’ - consider revising this sentence in order to enhance coherence between its first and second parts.

 ‘ *(…) Despite these barriers, some of them may be able to be overcome by addressing them in different ways as indicated below.*’ – the sentence serves as a conclusion to the entire section on barriers and should be placed in its own paragraph, further developed for clarity and depth.

*Facilitators of CST in Africa*

 ‘ *(…) Fourth, CST-SSA can be delivered by trained personnel.(…)*’ – the facilitator is that non-medical personnel can be trained, as stated above in the barriers section.

 ‘(…). *Participants often look forward’- * use the past tense.

 ‘(…) *Finally, the provision of transportation and refreshments was an important incentive for participation in the CST group sessions.*’ – provide reference.

*CST implementation in Africa*

*Recommendations*

 The recommendations presented earlier can be listed in this section.

 ‘(…) *It is aligned to the group therapy which supports the extended family system in Africa.’ – *this sentence needs further elaboration.

*Implications for practice*

 ‘(…) . *Group facilitators are to manage medical expectations on the part of the participants’ - *this sentence is repeating what has already been stated in the previous paragraphs.

Are the rationale for, and objectives of, the Systematic Review clearly stated?

Partly

Is the statistical analysis and its interpretation appropriate?

Not applicable

Are sufficient details of the methods and analysis provided to allow replication by others?

Partly

Are the conclusions drawn adequately supported by the results presented in the review?

Partly

Reviewer Expertise:

Cognitive neuroscience/experimental neuropsychology

I confirm that I have read this submission and believe that I have an appropriate level of expertise to confirm that it is of an acceptable scientific standard, however I have significant reservations, as outlined above.

# Reviewer report: Reviewer response for version 1

**DOI:** 10.21956/openresafrica.15249.r30373

**Published:** 2023-11-24

**Authors:** Diana Karamacoska

**Affiliations:** 1 NICM Health Research Institute, Western Sydney University, Penrith, Australia

Thank you for the opportunity to review this manuscript covering cognitive stimulation therapies for people living with dementia in Africa.  It was highly informative and of importance to dementia care and planning in this part of the world.  I have a few comments to improve the quality of the manuscript.

**Introduction:**

 The relevant literature was reviewed concisely and contextualised the paper well.

**Method:**

 Under the subheading “Design, protocol and registration”;

- In terms of the intervention design, you outline “the 14 sessions of CST include physical games, sound, childhood, food, current affairs, faces/senses, word association, being creative, categorizing objects, orientation, using money, number and word games, and team quiz”.  What does “sound, childhood, and food” actually refer to?  Can you describe these items better?

- Your outcomes (improvement in cognition, quality of life, and activities of daily living) would be more relevant to a systematic review and don’t seem to align with your objectives (1) study designs related to CST; 2) Adaptations/modifications in CST delivery in Africa; 3) barriers and facilitators to implementing CST within African contexts). Did you mean to say that papers were included in the scoping review if they measured CST’s impact on these outcome measures? Your scoping reviewing outcomes (and thus the data extracted from these papers) should reflect your objectives.

- Was your scoping review registered anywhere (such as the JBI website)? This information is missing from your Methods section.

Information about the database searches is repeated under the subheading “Selection of sources of evidence”. You can remove this information.

 In table 3, you have listed a systematic review by Stoner *et al.* (2021) as being included here. How is this possible when it is not a manuscript reporting on the outcomes of CST intervention? Furthermore, this systematic review would be reporting on the same CST papers that your scoping review has identified – I’d recommend clarifying your eligibility criteria and removing this paper from your analysis.

 Table 5 outcomes appear to report measures that go beyond what was expected/reported in the search criteria. Can you please refine this to match the purpose of the scoping review?  This table also reports patients as participant characteristics but it’s not clear what you mean by this – are they people living with dementia? If so, then please just use this terminology.

**Results:**

- “Six studies took place in Tanzania ^9, 22, 23, 25, 26, 28^ while four out of six studies were in Nigeria ^9, 25– 27^.” This does not make sense when you only included 7 papers and the locations shown in Table 5 list other countries.
- Usually the reporting of findings related to adaptability, feasibility, acceptability, delivery, outcomes, barriers and facilitators would be located in the results and not the discussion section.  I would recommend limiting your analysis and reporting to items that relate to your scoping review objectives.

**Discussion:**

- It’s unclear why there is commentary about India and Brazil when the scoping review is targeting African studies.
- Where did you get the data or evidence to support this claim “Places of worship are recommended to be avoided as these may cause acceptability challenges for those from other denominations or religions”? Can you please cite the relevant papers?
- Regarding feasibility, usually we would write about how this was assessed and then explain what the outcomes were from the studies. Why would funding be challenging for feasibility? Where did this information come from? If feasibility is being determined by attendance rates, how does its conduct by non-specialist staff relate to this?
- Acceptability is discussed in terms of costs. This would be relevant to participants if *they *had to pay for the service. Is this what happened in the studies? If not, then this usually would be regarded as an implementation cost so I would recommend reporting it in the relevant section.
- The “Content, delivery and implementation of CST in Africa” section is quite bare. It would make sense to move things like “conduct by non-specialist staff” and implementation costs here.
- Table 6 appears to replicate information from Table 5, in a synthesised format. I’d recommend removing table 6.

Overall, there is a lot of work that needs to be done to improve the rigour of this review.

Are the rationale for, and objectives of, the Systematic Review clearly stated?

Yes

Is the statistical analysis and its interpretation appropriate?

Partly

Are sufficient details of the methods and analysis provided to allow replication by others?

Partly

Are the conclusions drawn adequately supported by the results presented in the review?

Partly

Reviewer Expertise:

Dementia is my area of expertise and I have published several review articles, including integrative scoping and systematic reviews.

I confirm that I have read this submission and believe that I have an appropriate level of expertise to state that I do not consider it to be of an acceptable scientific standard, for reasons outlined above.
